# Investigating high schoolers’ L2 writing anxiety, L2 writing self-efficacy, L2 writing self-regulated strategies, and L2 writing engagement: Relationships and mediator

**DOI:** 10.3389/fpsyg.2022.1012407

**Published:** 2022-11-23

**Authors:** Jie Zhou, Shusheng Wang, Junju Wang

**Affiliations:** ^1^School of Foreign Languages and Literature, Shandong University, Jinan, China; ^2^School of Foreign Languages and Literature, Hunan University of Technology, Zhuzhou, China

**Keywords:** high school students, L2 writing anxiety, L2 writing self-efficacy, L2 writing self-regulated strategies, L2 writing engagement

## Abstract

This study used a structural equation modeling approach to investigate the relationships among L2 writing anxiety, L2 writing self-efficacy, L2 writing self-regulated strategies and L2 writing engagement, and possible mediators that regulate the effect of individual factors. A questionnaire was administered to 340 Chinese high school students from different parts of the country. The results of the study suggested a negative relationship between L2 writing anxiety and L2 writing self-efficacy, and a direct effect of both on L2 writing engagement. While a higher level of L2 writing self-efficacy indicated a lower level of L2 writing anxiety and more students’ L2 writing engagement, L2 writing efficacy had a much stronger direct effect on L2 writing engagement than L2 writing anxiety. In addition, L2 writing self-regulated strategies were found to play a mediating role between L2 writing self-efficacy and L2 writing engagement, but not between L2 writing anxiety and L2 writing engagement. This study helps to understand the interplay of individual factors related to L2 writing and sheds light on promoting English writing abilities of Chinese high school students.

## Introduction

Writing is a complex and versatile skill (Han and Hiver, 2018). Given its benefits for individuals to describe events, present information, and exchange opinions (Hayes, 2012), writing plays an important role in facilitating one’s success of study, job, and daily life (Graham et al., 2018). In pedagogical contexts, the development of writing ability is crucial for L2 learners since it is a key indicator that reflects on their overall language proficiency level (Teng et al., 2022).

Despite its increasing importance, L2 writing is viewed as a challenge for EFL learners due to its complicated and multifaceted nature, often influenced by the interplay of individual factors (Teng and Zhang, 2018). When writing, L2 learners often encounter various difficulties involving linguistic knowledge and writing skills which may result in writing anxiety, a lack of self-confidence (Cheng, 2004), and fear of negative evaluation and time limits (Kırmızı and Kırmızı, 2015). To a large extent, learner factors like anxiety and self-efficacy are determinant to students’ success in writing activities (Han and Hiver, 2018).

Apart from the negative influence of L2 writing anxiety, L2 writing is also affected by L2 writing self-efficacy in a positive way (Woodrow, 2011), and the interaction between writing anxiety and self-efficacy has an impact on the degree of L2 writing engagement (Zhou and Hiver, 2022). Given the need for self-regulation as a major aspect of self-efficacy (Bandura, 1997), learners who have a good perception of L2 writing self-regulated strategies tend to have a stronger sense of writing self-efficacy and demonstrate better writing performance (Teng and Zhang, 2018). In this sense, L2 writing self-regulated strategies are thought to play a crucial role in regulating emotions and achieving success in L2 writing (Teng et al., 2022).

In the Chinese context, high school students are studying in a fierce competitive environment, under the pressure of passing the National Entrance Examination for colleges and universities (Liu and Wang, 2021). For the English test where more weight is putting on writing performance, students have to work hard to pursue high marks in writing tasks. However, they are often confronted with various difficulties due to their poor knowledge of genres and undesirable language proficiency (Li, 2017), which consequently leads to their negative feelings and lack of self-efficacy. It is thus conductive to investigate the individual factors that affect Chinese high schoolers’ L2 writing and explore how these factors are related to one another and what mediates their effect on L2 writing engagement.

Previous studies on how L2 writing anxiety and L2 writing self-efficacy interact with each other were mostly focused on university students (e.g., Pajares, 2003; Woodrow, 2011; Kırmızı and Kırmızı, 2015; Abolhasani et al., 2022). Few studies have examined the interactions among L2 writing anxiety, L2 writing self-efficacy and L2 writing self-regulated strategies, and their influences on L2 writing engagement in the Chinese context, particularly in Chinese high schools. In view of this, this study takes Chinese high school students into consideration and examines the relationships among their L2 writing anxiety, L2 writing self-efficacy, L2 writing self-regulated strategies, and L2 writing engagement. It is hoped that this study provides insights for understanding the individual factors involved in Chinese high schoolers’ L2 writing, and help students further improve their well-being of L2 writing.

## Literature review

### L2 writing anxiety

L2 writing anxiety has been found to be significantly related to L2 writing performance (Cheng, 2002; Tahmouresi and Papi, 2021; Abolhasani et al., 2022). Consisting of three dimensions of cognitive anxiety, somatic anxiety and avoidance behavior, it refers to “a relatively stable anxiety disposition associated with writing, which involves a variety of dysfunctional thoughts, increased physiological arousal, and maladaptive behaviors” (Cheng, 2004, p.319). In addition, it is regarded as a situation-specific anxiety, since L2 writers might experience anxiety in various contexts, like in exams, in the class, at home, at work or in the community (Woodrow, 2011).

A large body of studies have found that L2 writing anxiety was negatively related to L2 writing self-efficacy (e.g., Pajares, 2003; Woodrow, 2011; Piniel and Csizér, 2013; Kırmızı and Kırmızı, 2015; Abolhasani et al., 2022). For example, a high level of writing anxiety may result in a decrease in writing self-efficacy (e.g., Kırmızı and Kırmızı, 2015), and students with a higher level writing self-efficacy experience a lower level of writing anxiety and have fewer writing avoidance behaviors (e.g., Pajares, 2003).

L2 writing anxiety is closely related to the use of L2 writing self-regulated strategies. Many studies demonstrated that students with low L2 writing anxiety could use more L2 writing self-regulated strategies (e.g., Asmari, 2013; Machida and Dalsky, 2014; Abolhasani et al., 2022; Bailey and Almusharraf, 2022). For example, Machida and Dalsky (2014) found that L2 writing strategies could help less anxious students more than those more anxious students.

In terms of the relationship between L2 writing anxiety and L2 writing engagement, there have been discrepancies among researchers. Whereas some of them (e.g., Tsao et al., 2017) thought that a high level of anxiety would interfere with students’ engagement in the writing process, others (e.g., Astrid et al., 2017) argued that many students with low writing anxiety had positive behavior engagement and some students with high writing anxiety had positive emotional engagement in writing activities.

### L2 writing self-efficacy

L2 writing self-efficacy refers to L2 writers’ confidence and beliefs in their abilities to successfully perform writing tasks (Han and Hiver, 2018). Considered as the most reliable predictor of students’ writing performance (Bandura, 1997), it is believed to have a recursive relationship with L2 writing performance (Robinson et al., 2020), which means that L2 writing self-efficacy enhances L2 writing performance, and in turn, nurtures the development of L2 writing self-efficacy (Sun et al., 2021).

Inconsistent research findings exist concerning the relationship between L2 writing self-efficacy and L2 writing self-regulated strategies. Whereas many studies suggested a positive influence of L2 writing self-efficacy on the use of L2 writing self-regulated strategies (e.g., Zimmerman and Risemberg, 1997; Pajares, 2003; Bruning et al., 2013; Teng and Huang, 2019; Sun and Wang, 2020), some found that there is no significant relationship between L2 writing self-efficacy and L2 writing self-regulated strategies (e.g., Graham et al., 2005). This might be associated with the inaccuracies of assessing the capabilities for young children by themselves.

In the meantime, some studies found that L2 writing self-efficacy was positively related to writing engagement (e.g., Usher and Pajares, 2008; Han and Hiver, 2018; Tsao, 2021). For example, Usher and Pajares (2008) found that L2 writing self-efficacy could enhance students’ engagement in the writing process. Han and Hiver (2018) pointed out that L2 writing self-efficacy could moderate attention and cognitive engagement, and determine the level of effort that students would put into L2 writing activities. Tsao (2021) also reported that L2 writing self-efficacy was a predictive power to motivate students to engage in different types of written corrective feedback.

### L2 writing self-regulated strategies

According to Teng and Zhang (2016), L2 writing self-regulated strategies are students’ “deliberate, goal-directed attempts to make writing enjoyable, less challenging, and more effective” (p. 7). In the multidimensional model that they conceptualized, L2 writing self-regulated strategies included cognitive strategies, metacognitive strategies, social-behavioral strategies, and motivational regulation strategies.

Previous studies reported that L2 writing self-regulated strategies play a facilitative role for successful L2 writing (e.g., Teng and Zhang, 2016; Teng and Reynolds, 2019; Teng et al., 2022), and are closely linked to how L2 writers monitor their performance and adjust their tasks to achieve success (Zimmerman, 2001; Han and Hiver, 2018). A meta-analysis by Santangelo et al. (2016) also demonstrated that the use of writing self-regulated strategies contributed significantly to the improvement of students’ writing performance.

Previous studies also suggested that L2 writing self-regulated strategies was positively related to L2 writing self-efficacy (Zimmerman and Risemberg, 1997; Ekholm et al., 2015), and was positively associated with L2 writing engagement (Zhou and Hiver, 2022). Csizèr and Tankó (2017), for example, found that self-regulated strategies could increase writing self-efficacy and decrease writing anxiety for L2 writers. Teng and Zhang’s (2018) study also revealed that L2 writing self-regulated strategies mediated the effect of motivational regulation strategies on L2 writing performance.

### L2 writing engagement

L2 writing engagement refers to students’ active and productive involvement in writing activities (Reeve et al., 2020). It is conceptualized as a multidimensional construct comprising behavioral, cognitive, emotional facets (Fredricks et al., 2004) and agentive engagement (Reeve and Tseng, 2011).

Some studies demonstrated that L2 writing engagement was positively linked to students’ writing performance (e.g., Qi and Lapkin, 2001; Fredricks et al., 2004; Rahimi and Zhang, 2021). Qi and Lapkin (2001), for instance, found that the extensiveness of students’ engagement and the quality of notice may lead to improved writing performance. Rahimi and Zhang’s (2021) study suggested that students’ experiences with the engaging process-genre approach to writing were found to assist each other in sustaining engagement and achievements in and beyond the classroom.

Few studies have suggested that L2 writing engagement was related to some other psychological factors (e.g., Han and Hiver, 2018; Zhou and Hiver, 2022). It was noted that L2 writing self-regulated strategies functioned as an important predictor to students’ L2 writing engagement in the writing class (Zhou and Hiver, 2022).

### Research hypotheses

In view of the aforementioned studies, it is quite clear that most of them attempted to investigate the relationship between a certain factor and writing performance, and very few have dealt with two or three individual factors, with scarce attention paid to L2 writing engagement. Despite the abundant findings on L2 writing anxiety, L2 writing self-efficacy, and L2 writing self-regulated strategies, inconsistent findings have been yielded and the relationships among the three factors were largely underexplored. Besides, previous studies have predominantly focused on university students in other countries than the Chinese context, little is known about Chinese students, particularly Chinese high school students.

**Figure 1:**
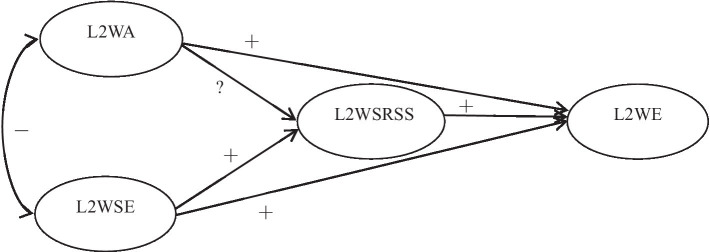
A hypothesized model of the relationship among the four variables.

Given the above limitations, this study aims to focus on Chinese high school students and investigate the relationships among L2 writing anxiety, L2 writing self-efficacy, L2 writing self-regulated strategies, and L2 writing engagement. To achieve such a goal, a hypothesized model was developed (see Figure 1), in which L2 writing anxiety (L2WA) and L2 writing self-efficacy (L2WSE) were the predictors, L2 writing self-regulated strategies (L2WSRSS) a mediator, and L2 writing engagement (L2WE) the dependent variable. It was hypothesized that L2 writing self-regulated strategies can mediate the effects of L2 writing anxiety and L2 writing self-efficacy on L2 writing engagement.

Based on the above model, the following hypotheses are to be tested.H1:L2 writing anxiety is negatively associated with L2 writing self-efficacy; both L2 writing anxiety and L2 writing self-efficacy have a positive direct effect on L2 writing engagement.H2:L2 writing self-efficacy is positively associated with L2 writing self-regulated strategies and L2 writing engagement.H3:L2 writing self-regulated strategies are positively related to L2 writing engagement and mediate the effects of L2 writing anxiety and L2 writing self-efficacy on L2 writing engagement.

## Materials and methods

### Participants

A total of 340 students from 20 Chinese high schools participated in this study. These students came from both key and regular high schools in eight provinces across the country, including the eastern, western, southern, and northern regions of China. Among them, 128 were from key high schools and 212 came from regular high schools. Aged from 14 to 20 years old (*M* = 16.06, *SD* = 1.45), this group of students included 157 boys and 183 girls, with 226 of them in their first year, 33 in their second year, and 81 were in their third year. When the survey was conducted, these students had been attending high school English classes from 6 months to 30 months. As the overwhelming majority of their peers, their English studies had been following the same curriculum prescribed by the Chinese Ministry of Education. Each week, they had six class hours for English learning. Quizzes were regularly administered on weekly and monthly basis. Typically, they took one mid-term and one final-term English tests which basically followed the norms and requirements of the National College Entrance Examination. They were all Chinese native speakers and had been learning English as their compulsory subject since their primary education. English was their only foreign language and none of them had studied overseas or traveled to other countries.

### Instruments

A composite questionnaire (See the Appendix) was used in this study to investigate Chinese high school students’ L2 writing anxiety, L2 writing self-efficacy, L2 writing self-regulated strategies, and L2 writing engagement. The first part of the questionnaire collected the demographic information of the participants. The second part of the questionnaire, consisting of 19 items, was made up of four subscales rated on a five-point Likert scale, ranging from 1 (strongly disagree) to 5 (strongly agree).

The L2 Writing Anxiety Scale (L2WAS) was adapted from Cheng’s (2004) Second Language Writing Anxiety Inventory to measure L2 learners’ writing anxiety. It contained four items covering cognitive anxiety, somatic anxiety, and avoidance behavior anxiety. A sample item is “I am afraid that my English composition will be chosen for class discussion or evaluation.” The internal consistency (Cronbach’s *α* coefficient) of the subscale was 0.817.

The L2 Writing Self-Efficacy Scale (L2WSES) was adapted from Wang and Bai’s (2017) Questionnaire of English Self-Efficacy and Sun and Wang’s (2020) Questionnaire of English Writing Self-Efficacy. The subscale consisted of five items in five categories, namely organization, use of English writing, ideation, grammar and spelling, and self-efficacy for self-regulation. A sample item is “I can organize sentences into a paragraph to express an idea.” The internal consistency (Cronbach’s *α* coefficient) of the subscale was 0.890.

The L2 Writing Self-Regulated Strategy Scale (L2WSRSS) was adapted from the Self-Regulatory Writing Strategy Questionnaire by Teng et al. (2022) to measure the use of writing self-regulated strategies. This subscale consisted of four items covering cognitive strategy, social-behavioral strategy, motivational regulation strategy, and metacognitive strategy, following Teng and Zhang (2016) model. A sample item is “I believe that studying writing strategies will lead to better writing performance.” The internal consistency (Cronbach’s *α* coefficient) of the subscale was 0.897.

The L2 Writing Engagement Scale (L2WES) was adapted from Martin’s (2012) English Writing Motivation and Engagement Scale to assess students’ L2 writing motivation and engagement. This subscale consisted of six items covering emotional (2 items), cognitive (2 items), and behavioral engagement (2 items). A sample item is “When I encounter difficulties in the writing process, I try to overcome them.” The internal consistency (Cronbach’s *α* coefficient) of this subscale was 0.837.

### Procedure and data analysis

Data collection of this study was done in January 2022 with the help of the headteachers of the participants. As most participants were teenagers, a consent letter was sent to their parents or legal guardians *via* text messages or WeChat messages. After getting their parents’ or legal guardians’ consents, the headteachers helped administer the questionnaire to the participants *via* an online link sent to students’ WeChat or QQ class groups. Prior to that, the participants were informed of the purposes of the study, their roles in data collection, and the confidential and voluntary nature of the study. They were asked to sign on the questionnaire to ensure their willingness to join the study. Three weeks later, 365 copies of responded questionnaire were collected and 340 of them were found valid, with a 93.15% response rate.

When analyzing the collected data, we adopted AMOS 25.0 and SPSS 26.0 to test the hypotheses by conducting confirmatory factor analysis (CFA) on the validity and examining the reliability of the subscales. Descriptive analysis was done for frequencies, means, and standard deviations. Pearson’s correlation coefficients were calculated for the relationship among L2 writing anxiety, L2 writing self-efficacy, and L2 writing engagement. Structural equation modeling was for the analysis of the path relationship among variables. Bootstrapping was utilized to assess the mediation effect of L2 writing self-regulated strategies.

The goodness-of-fit statistics included chi-square statistics (*χ^2^*), degrees of freedom (*df*), *p*-value, root-mean-square error of approximation (RMSEA), comparative fit index (CFI), and Tracker-Lewis index (TLI). In terms of model fitness judgment, it is generally considered that the model fitness is reasonable when the evaluation indexes of SEM fitness meet the following criteria: CFI > 0.90, TLI > 0.90, RMSEA<0.10 (Byrne, 2016). A significant *p*-value indicates that the model may be appropriate. The guidelines of Gignac and Szodorai (2016) were adopted to interpret the effect size, by which small = 0.10–0.20, medium = 0.20–0.30, and large = ≥0.30.

## Results

### Validity and reliability

Results of the study indicate that the four subscales had high structural validity and reliability and all four subscales offered an acceptable fit to the data.

On the basis of Maximum likelihood estimation, CFA was conducted through AMOS 25.0 to assess the overall fitting degree of the four subscales (L2WAS, L2WSES, L2WSRSS, and L2WES) before the hypothesized model was examined. As long as most indexes reach the standard, the data and model fitting can be identified. The fitting indexes of the model all reached the standard of good model fitness (*χ^2^* = 362.84, *df* = 146, *p* < 0.001, CFI = 0.94, TLI = 0.93, RMSEA = 0.066).

As presented in Table 1, it is identified that the four-factor model of L2WAS fit well (*χ^2^* = 8.29, *df* = 2, *p* < 0.001, TLI = 0.96, CFI = 0.98, RMSEA = 0.096) based on the fit indices criteria (Byrne, 2006); the five-factor model of L2WSES fit adequately (*χ^2^* = 31.48, *df* = 5, *p* < 0.001, TLI =0.94, CFI = 0.97, RMSEA = 0.125); the four-factor model of L2WSRSS fit as well (*χ^2^* = 6.95, *df* = 2, *p* < 0.001, TLI = 0.98, CFI = 0.99, RMSEA = 0.085); the six-factor model of L2WES also fit greatly (*χ^2^* = 17.21, *df* = 9, *p* < 0.001, TLI = 0.98, CFI = 0.99, RMSEA = 0.052). The factor loadings of the four subscales ranged from.64 to 0.80 (L2 writing anxiety), 0.67 to 0.84 (L2 writing self-efficacy), 0.77 to 0.87 (L2 writing self-regulated strategies), and 0.56 to 0.82 (L2 writing engagement).

**Table 1:** The fitting indexes and coefficients of the model.

	*χ^2^*	*df*	*p*	TLI	CFI	RMSEA	Coefficient *α*
L2WAS	8.29	2	0.00	0.96	0.98	0.096	0.82
L2WSES	31.48	5	0.00	0.94	0.97	0.125	0.89
L2WSRSS	6.95	2	0.00	0.98	0.99	0.085	0.90
L2WES	17.21	9	0.00	0.98	0.99	0.052	0.84

In addition, the discriminant validity was tested by comparing the square root of AVE for each subscale and correlation coefficients between each pair of subscales. As Table 1 shows, it is evident that for each subscale the square root of AVE is larger than the correlation coefficients, showing good discriminant validity.

### Descriptive statistics and correlations

Results of descriptive analysis (see Table 2) show that of the four variables, the mean value of L2 writing engagement was the highest (*M* = 7.66, *SD* = 3.82), followed by L2 writing self-efficacy (*M* = 15.12, *SD* = 4.57), then L2 writing anxiety (*M* = 3.26, *SD* = 4.26), and finally L2 writing self-regulated strategies (*M* = 12.02, *SD* = 3.90).

**Table 2:** Descriptive statistics, correlations, and reliability (*N* = 340).

	L2WAS	L2WSES	L2WSRSS	L2WES	Factor Loadings
L2WAS	**0.82**				0.64–0.80
L2WSES	−0.07	**0.89**			0.67–0.84
L2WSRSS	−0.06	0.75^**^	**0.90**		0.77–0.87
L2WES	0.01	0.66^**^	0.71^**^	**0.84**	0.56–0.82
Mean	13.26	15.12	12.02	17.66	
Standard Deviation	4.26	4.57	3.90	3.82	

**p < 0.01; Cronbach’s *α* coefficients are in bold on diagonals.

Results of correlation analysis revealed a negative relationship between L2 writing anxiety and L2 writing self-regulated strategies (*r* = −0.06, *p* > 0.05) and a positive relationship between L2 writing anxiety and L2 writing engagement (*r* = 0.01, *p* > 0.05), despite not significant. In the meantime, it is identified that L2 writing self-efficacy was found to have a strong positive relationship with L2 writing self-regulated strategies (*r* = 0.75, *p* < 0.001), and L2 writing engagement (*r* = 0.66, *p* < 0.001) and that L2 writing self-regulated strategies had a highly positive relationship with L2 writing engagement (*r* = 0.71, *p* < 0.001).

### Structural equation modeling analysis

**Figure 2:**
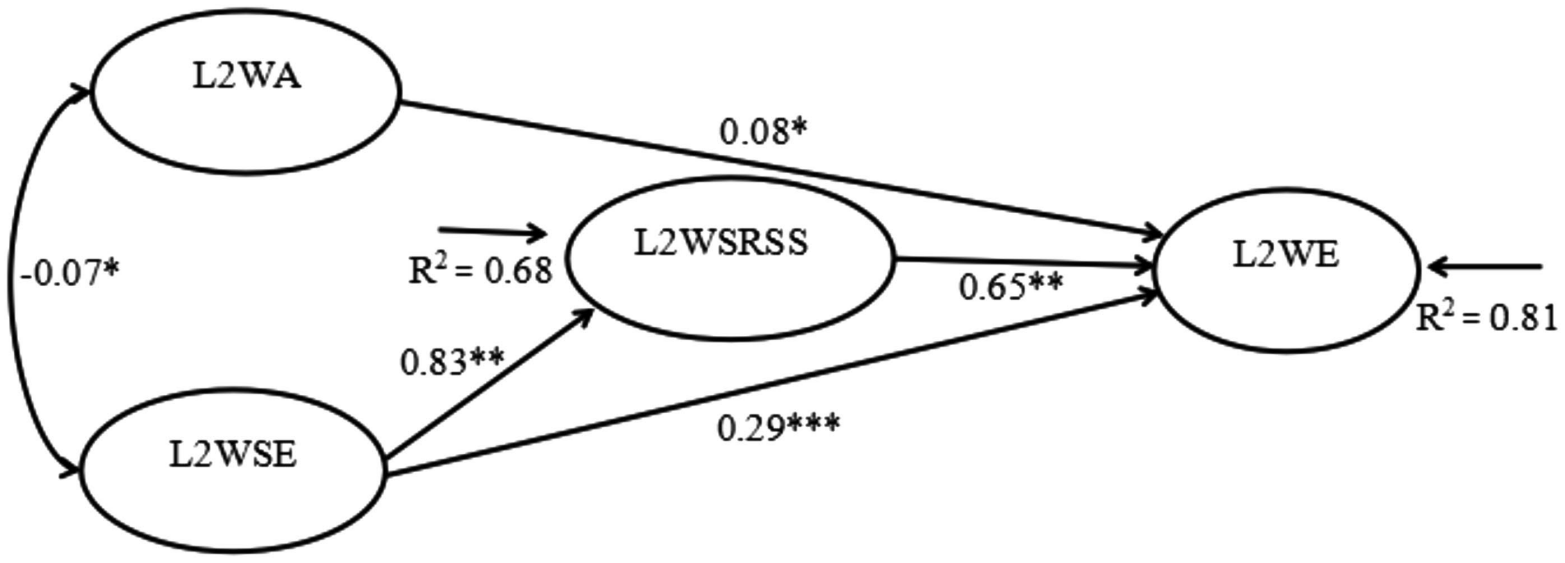
Results of SEM model (*N* = 340). ***p < 0.001, *p < 0.05; goodness-of-fit indices: *χ^2^* = 362.84, *df* = 146, *p < 0*.001, CFI = 0.94, TLI = 0.93, RMSEA =0.066; non-statistically significantly paths (*p* ≧ 0.05) are not reported.

Figure 2 shows the coefficients of the final model and its standardized path. Although the relationship between L2 writing anxiety and L2 writing self-efficacy was not significant, L2 writing anxiety was found negatively associated with L2 writing self-regulated strategies (*β* = −0.01, *p* < 0.05) and positively related to L2 writing engagement (*β* = 0.08, *p* < 0.05), supporting Hypothesis 1. Besides, it was found that L2 writing self-efficacy was positively related to L2 writing self-regulated strategies (*β* = 0.83, *p* < 0.001) and L2 writing engagement (*β* = 0.29, *p* < 0.001) and that L2 writing self-regulated strategies had a positive relationship with L2 writing engagement (*β* = 0.65, *p* < 0.001), supporting Hypothesis 2.

### Mediation analysis

Based on Preacher and Hayes (2008), bootstrapping was utilized on the basis of 5,000 samples to examine the mediation effect of L2 writing self-regulated strategies. If the 95% confidence interval between the lower and upper bound did not include zero, indirect effects would be significant (Hayes, 2009).

The mediation effect of L2 writing self-regulated strategies between L2 writing anxiety and L2 writing engagement showed very small effect size (<|0.10|) whereas the mediation effect of L2 writing self-regulated strategies between L2 writing self-efficacy and L2 writing engagement showed large effect size (≧|0.30|; see Gignac and Szodorai, 2016). As Figure 2 shows, the 95% confidence interval of indirect effect between L2 writing self-efficacy and L2 writing engagement did not include zero [0.42, 0.69], indicating that L2 writing self-regulated strategies significantly mediated the relationship between L2 writing self-efficacy and L2 writing engagement (*β* = 0.53, *p* < 0.001). However, the 95% confidence interval of indirect effect between L2 writing anxiety and L2 writing engagement included zero [−0.074, −0.04], suggesting that L2 writing self-regulated strategies did not mediate the relationship between L2 writing anxiety and L2 writing engagement. Thus, Hypothesis 3 was rejected.

## Discussion

### Direct effect

Results of SEM analysis showed that there was a significant negative relationship between L2 writing anxiety and L2 writing self-efficacy, but the magnitude of the correlation was small. The significant negative relationship between anxiety and self-efficacy in L2 writing was consistent with previous findings (e.g., Woodrow, 2011; Piniel and Csizér, 2013; Kırmızı and Kırmızı, 2015), which suggested that learners with high levels of writing self-efficacy experienced low levels of L2 writing anxiety, and vice versa. Since writing is usually the most challenging part of English exams, it can put more pressure on high school students and hence make them more likely to be overwhelmed by negative feelings (Liu and Wang, 2021). Possibly, the high levels of L2 writing anxiety that the participants of this study experienced was caused by the fear of getting a low grade or negative evaluation. These stressful thoughts were unrelated to the actual writing tasks, and excessively occupied their limited cognitive resources, thus negatively affecting their writing self-efficacy.

Different from previous studies that focused on the self-efficacy in the development of L2 writing, this study focused on high school students’ self-efficacy in their writing tasks based on the contents of the questionnaire. Based on the results of this study, it is clear that students’ levels of writing self-efficacy were higher than their levels of writing anxiety. Such a discrepancy was possibly associated with the specific context of Chinese high school students’ L2 writing tasks. In many cases, they wrote for exams and experienced various types of pressure, particularly that from the highly competitive National College Entrance Examination (Kirkpatrick and Zang, 2011). In addition, mostly lacking systematic training of L2 writing, they tended to rely more on memorizing certain knowledge and materials (Teng et al., 2022).

The study also found L2 writing self-efficacy had a significant positive effect on L2 writing engagement, suggesting that students with higher L2 writing self-efficacy would have high degree of L2 writing engagement. Similarly, Hetthong and Teo (2013) found students with higher L2 writing self-efficacy would regard difficulties as tasks to be mastered, form commitment, develop their interests, and generate more efforts to enhance their L2 writing engagement. This result confirmed the positive effect of L2 writing self-efficacy on writing performance and writing motivation (Sun et al., 2021), which means that higher levels of writing self-efficacy could stimulate students’ achievement motivation, guide them to actively engage in the writing process, and result in better writing performance (Teng and Zhang, 2018).

Additionally, the above findings may be attributed to the newly adopted teaching approaches in Chinese schools such as genre-oriented approach, process-oriented approach and collaborative writing (Yu and Lee, 2016). New teaching methods put more emphasis on learner autonomy and cooperative learning and may have influence on students’ self-efficacy and promote their engagement in L2 writing. According to Bandura’s (1997) social cognitive theory, students with higher L2 writing self-efficacy tended to make use of L2 writing self-regulated strategies to engage in the writing activities.

### The mediating effect

Results of SEM analysis demonstrated that L2 writing self-regulated strategies played a significant mediating role between L2 writing self-efficacy and L2 writing engagement. The low-level direct effect between self-efficacy and engagement in L2 writing indicated that L2 writing self-efficacy could not completely regulate the degree of L2 writing engagement. However, L2 writing self-regulated strategies did not mediate between L2 writing anxiety and L2 writing engagement.

Of all these factors, L2 writing self-efficacy had the strongest positive effect on the L2 writing self-regulated strategies, and L2 writing self-regulated strategies had the second strongest positive effect on the L2 writing engagement. It further confirmed that L2 writing self-regulated strategy was considered as an important indicator of students’ L2 writing engagement (Fredricks et al., 2004). The more self-efficacy students have, the more L2 writing self-regulated strategies they adopt, and the higher degree of writing engagement they involve.

The path coefficient suggested that L2 writing self-efficacy was highly positively related to L2 writing self-regulated strategies. This supported the findings of previous studies (Yu et al., 2019; Sun and Wang, 2020), which implied that students with higher writing self-efficacy are usually motivated to adopt various writing self-regulated strategies to engage actively in L2 writing. However, the reduced effect size of L2 writing self-regulated strategies on L2 writing engagement indicated that Chinese high school students were not very strong in adopting more self-regulated strategies to enhance their engagement in L2 writing activities. This can be attributed to the lack of a systematic design of L2 writing courses, sufficient time on writing instruction, and a sustainable L2 writing assessment system for Chinese high school students (Ai, 2015). In addition, Chinese high school students might focus more on linguistic knowledge instead of practical use of writing under the exam-oriented educational system (Sun and Wang, 2020).

As indicated by Teng and Zhang (2018), students tended to feel more self-efficacious to engage in the L2 writing activities with an increase in using L2 writing strategies. In this sense, L2 writing self-regulated strategies could predict L2 writing engagement for enhancing writing performance since self-regulated students can more easily reflect on their work, process teachers or peers feedback, and set goals to manage and evaluate their writing performance (Teng and Huang, 2019). According to the cognitive process theory (Hayes, 2000), writing is a process-oriented communicative activity involving affective, cognitive, physical, and social conditions and factors such as L2 writing anxiety, L2 writing self-efficacy, L2 writing self-regulated strategies, and L2 writing engagement are all closely linked to the success of L2 writing. This explains why the combined use of cognitive, metacognitive, motivational, and social behavioral strategies can promote the quantity and quality of their engagement in the writing process.

## Conclusion

In this study, we explored the relationships among L2 writing anxiety, L2 writing self-efficacy, L2 writing self-regulated strategies, and L2 writing engagement in the Chinese high school context. It has been found that there was a negative relationship between L2 writing anxiety and L2 writing self-efficacy, and the higher level of L2 writing self-efficacy, the lower level of L2 writing anxiety. Both L2 writing anxiety and L2 writing self-efficacy had a direct effect on students’ L2 writing engagement, with the direct effect of L2 writing efficacy being much higher that of L2 writing anxiety. L2 writing self-regulated strategies played a mediating role between L2 writing self-efficacy and L2 writing engagement, and this mediating effect compensated for the insufficient effect of L2 writing self-efficacy on L2 writing engagement for high school students.

## Implications and limitations

The findings of this study suggest some pedagogical implications. Given the negative relationship between L2 writing anxiety and L2 writing self-efficacy, as found in this study, teachers are suggested take various measures to provide more positive feedback on L2 writing, offer more encouragement to foster students’ agency, and enhance students’ L2 writing self-efficacy to alleviate the negative impact of L2 writing anxiety. Given the mediating role of L2 writing self-regulated strategies, it is desirable for teachers to strengthen the training of students’ L2 writing self-regulated strategies, enhance their writing engagement, and improve their L2 writing techniques to cope with various difficult challenges in L2 writing. Besides, teachers are also encouraged to help students establish specific L2 writing goals and guide then engage actively and agentively in writing activities so as to improve their writing performance.

There are still some limitations of this study. First, the present study is independent of the specific writing tasks, so it is difficult to demonstrate the interaction between L2 writing anxiety and L2 writing self-efficacy. Second, although the study revealed the mediating role of L2 writing self-regulated strategies, it did not clarify what specific L2 writing self-regulated strategies could play such a role. Finally, given the huge number of Chinese high schoolers, the sample size of this study is not large enough to reflect the overall situation of Chinese high school students. In view of this, future studies could enlarge samples of learners to investigate students’ L2 writing self-efficacy and emotion-regulation in specific writing tasks. Qualitative methods such as interview and reflective journal could be integrated with questionnaire survey to explore the implementation of L2 writing self-regulated strategies in the writing process.

## Data availability statement

The original contributions presented in the study are included in the article/supplementary material, further inquiries can be directed to the corresponding author.

## Ethics statement

The studies involving human participants were reviewed and approved by the School of Foreign Languages and Literature, Shandong University, China. Written informed consent to participate in this study was provided by the participants and their legal guardians/next of kin.

## Author contributions

JZ collected the data and wrote the first draft of the manuscript. SW analyzed the data. JZ and JW revised the manuscript. All the authors contributed to the final round of revision and approved the submitted version.

## Funding

This work was supported as a Major Project of the Chinese National Social Science Foundation (grant no. 17AYY022).

## Conflict of interest

The authors declare that the research was conducted in the absence of any commercial or financial relationships that could be construed as a potential conflict of interest.

## Publisher’s note

All claims expressed in this article are solely those of the authors and do not necessarily represent those of their affiliated organizations, or those of the publisher, the editors and the reviewers. Any product that may be evaluated in this article, or claim that may be made by its manufacturer, is not guaranteed or endorsed by the publisher.
